# *Trichomonas vaginalis* Exosomes Deliver Cargo to Host Cells and Mediate Host∶Parasite Interactions

**DOI:** 10.1371/journal.ppat.1003482

**Published:** 2013-07-11

**Authors:** Olivia Twu, Natalia de Miguel, Gila Lustig, Grant C. Stevens, Ajay A. Vashisht, James A. Wohlschlegel, Patricia J. Johnson

**Affiliations:** 1 Molecular Biology Institute, University of California, Los Angeles, California, United States of America; 2 Department of Microbiology, Immunology and Molecular Genetics, University of California, Los Angeles, California, United States of America; 3 IIB-INTECH, CONICET-UNSAM, Camino de Circunvalación Laguna Km. 6, Buenos Aires, Argentina; 4 Department of Biological Chemistry, University of California, Los Angeles, California, United States of America; University of Virginia Health System, United States of America

## Abstract

*Trichomonas vaginalis* is a common sexually transmitted parasite that colonizes the human urogential tract where it remains extracellular and adheres to epithelial cells. Infections range from asymptomatic to highly inflammatory, depending on the host and the parasite strain. Here, we use a combination of methodologies including cell fractionation, immunofluorescence and electron microscopy, RNA, proteomic and cytokine analyses and cell adherence assays to examine pathogenic properties of *T. vaginalis*. We have found that *T.vaginalis* produces and secretes microvesicles with physical and biochemical properties similar to mammalian exosomes. The parasite-derived exosomes are characterized by the presence of RNA and core, conserved exosomal proteins as well as parasite-specific proteins. We demonstrate that *T. vaginalis* exosomes fuse with and deliver their contents to host cells and modulate host cell immune responses. Moreover, exosomes from highly adherent parasite strains increase the adherence of poorly adherent parasites to vaginal and prostate epithelial cells. In contrast, exosomes from poorly adherent strains had no measurable effect on parasite adherence. Exosomes from parasite strains that preferentially bind prostate cells increased binding of parasites to these cells relative to vaginal cells. In addition to establishing that parasite exosomes act to modulate host∶parasite interactions, these studies are the first to reveal a potential role for exosomes in promoting parasite∶parasite communication and host cell colonization.

## Author Summary

Trichomoniasis, the most common non-viral sexually transmitted disease worldwide, infects over 275 million people annually. Infection results from the colonization of the human urogenital tract by the parasite *Trichomonas vaginalis*. To establish and maintain infection the parasite adheres to host cells, a process that is poorly understood. Here, we show that *T. vaginalis* secretes small vesicles called exosomes that are capable of fusing with and delivering their contents to host cells. Parasite exosomes were found to induce changes in the host cell and to mediate the interaction of *T. vaginalis* with host by increasing the adherence of the parasite to host cells. Exosomes have been primarily studied in mammalian cells where they have been shown to mediate intercellular communication and have been implicated in processes including development, antigen presentation and cancer metastasis. Our data extend the function of exosomes to mediating host∶parasite interactions, cellular communication between two species and promoting colonization of an extracellular parasite. Research on *T. vaginalis* exosomes holds the potential for developing applications that would allow exosomes to be used in detecting and diagnosing trichomoniasis and for targeting drugs to the site of infection.

## Introduction

The parasite *Trichomonas vaginalis* causes the most common non-viral sexually transmitted infection, with an estimated 275 million people infected each year worldwide [1]. Disease manifestations may include vaginitis, cervicitis, urethritis, pelvic inflammatory disease, and adverse birth outcomes [2]. *T.vaginalis* infection may also increase risk of HIV transmission and the incidence and severity of cervical and prostate cancer [3]–[5]. Despite the need to define key pathogenic properties of the parasite in order to prevent and control the infection, little is known about parasite or host factors involved in pathogenesis [6], [7]

As an extracellular parasite residing in the urogenital tract, *T. vaginalis* must adhere to epithelial cells as an initial step towards colonizing the host and establishing infection. Although several families of membrane proteins and secreted proteases have been proposed to play roles in host cell attachment [7]–[10] only three *T.vaginalis* surface molecules have been shown to be involved in attachment of the parasite to host epithelial cells. The best studied is an abundant lipoglycan (TvLG) [11]–[13] that binds to galectin-1, the only host cell receptor described for *T.vaginalis* [14]. Two related surface proteins of unknown function are known to increase the attachment of *T.vaginalis* to host cells when expressed in the parasite [15].

A recent analysis of the surface membrane proteome of *T.vaginalis* revealed that at least three tetraspanin (Tsp) proteins of the nine found in the genome are present on the parasite's surface [15], [16]. Tsps are involved in a wide variety of activities in mammalian cells including attachment, fusion, motility, migration, and proliferation [17]. Of the 33 human tetraspanins, a small subset including CD63, CD9, CD81, CD82 are constitutive components of exosomes and various other tetraspanins may be present depending on cell of origin and cellular environment [18]. Tsps are also present in all examined mammalian exosomes and, as such, are routinely used as markers for these small secreted extracellular vesicles [19], [20].

Exosomes are 30–100 nm membrane-bound vesicles derived from endocytic compartments that are secreted into the extracellular milieu. Studies of mammalian cells have established that exosomes package specific cargo used for intercellular communication [21], immune modulation and surveillance and the metastasis of diverse tumor cells [18], [22]–[24]. Roles in antigen presentation, the delivery of surface receptors and the transfer of RNA to recipient cells have also been described for exosomes [25]–[27].

Exosomes have recently been shown to be released by pathogens or mammalian cells infected with pathogens [22], [28]. However, to date only a handful of non-mammalian cell types: the fungi *Histoplasma*, *Cryptococcus*, *Paracoccidiodes*, the nematode *C.elegans* and the parasite *Leishmania* [29]–[31] have been shown to produce exosomes.

Here we report that *T.vaginalis* secretes exosomes with physical characteristics and protein components similar to mammalian exosomes. Our analyses demonstrate that parasite exosomes mediate both host∶parasite and parasite∶parasite interactions and play a role in the attachment of the parasite to host epithelial cells. *T. vaginalis* exosomes are also shown to fuse with and deliver their contents to host cells thereby modulating host cell immune response. These studies are the first to indicate a role for exosomes in promoting host cell colonization and parasite∶parasite communication.

## Results

### Accumulation of a *T.vaginalis* tetraspanin protein in vesicular bodies upon contact of the parasite with host cells

**Figure 1:**
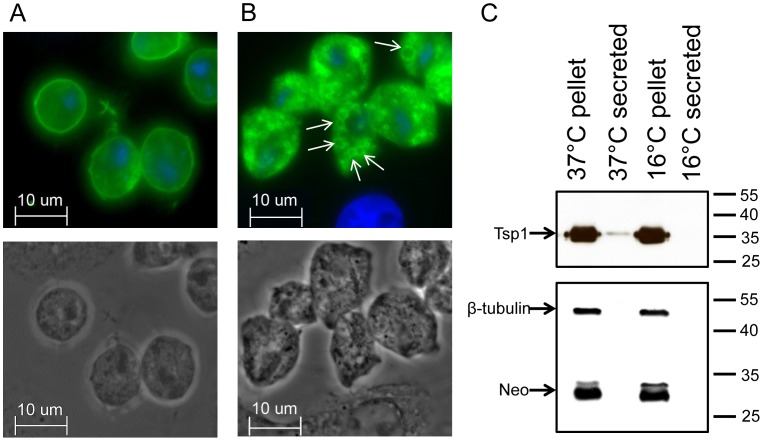
TSP1, a secreted protein, accumulates on large vesicles upon prolonged binding of *T.vaginalis* to Ects. *T. vaginalis* transfected with hemagglutin (HA) tagged TSP1 (Tvag_019180, green) in the presence of host ectocervical cells (Ects) for 15 min (A) or 60 min (B). Large vesicular structures containing Tsp1 that accumulate after 60 min are marked by arrows. Nuclei are labeled blue by DAPI stain. (C) Western blot analyses of supernatant and pelleted fractions harvested from *T. vaginalis* after incubation at 37°C or 16°C for 2 hrs. The blot was reacted with anti-HA antibodies to detect HA-tagged Tsp1 (∼35 kDa), anti β-tubulin (∼50 kDa) and anti neomycin phophotransferase (∼30 kDa) antibodies. Incubation at 16°C and detection β-tubulin and neomycin phophotransferase serve as negative controls for cell lysis.

As previously published in the surface proteome, three tetraspanin (Tsp) membrane proteins were identified [15], [16]. Examination of exogenously expressed, hemagglutinin (HA) tagged Tvag_019180 (Tsp1) revealed it is mainly on the plasma membrane (Fig. 1A) [15]. However, when parasites expressing Tsp1-HA are exposed for an hour or longer to ectocervical cells (Ects) the protein accumulates in large vesicular bodies within the parasite (Fig. 1B). These structures are reminiscent of mammalian multivesicular bodies that give rise to secreted exosomes. As mammalian Tsps are enriched in exosomes [19], [26], these data raised the possibility that *T. vaginalis* secretes Tsp-containing vesicles. As shown in Fig. 1C, this membrane protein is secreted, consistent with the secretion of exosomes by *T. vaginalis*.

### *T. vaginalis* secretes vesicles with characteristics of exosomes

**Figure 2:**
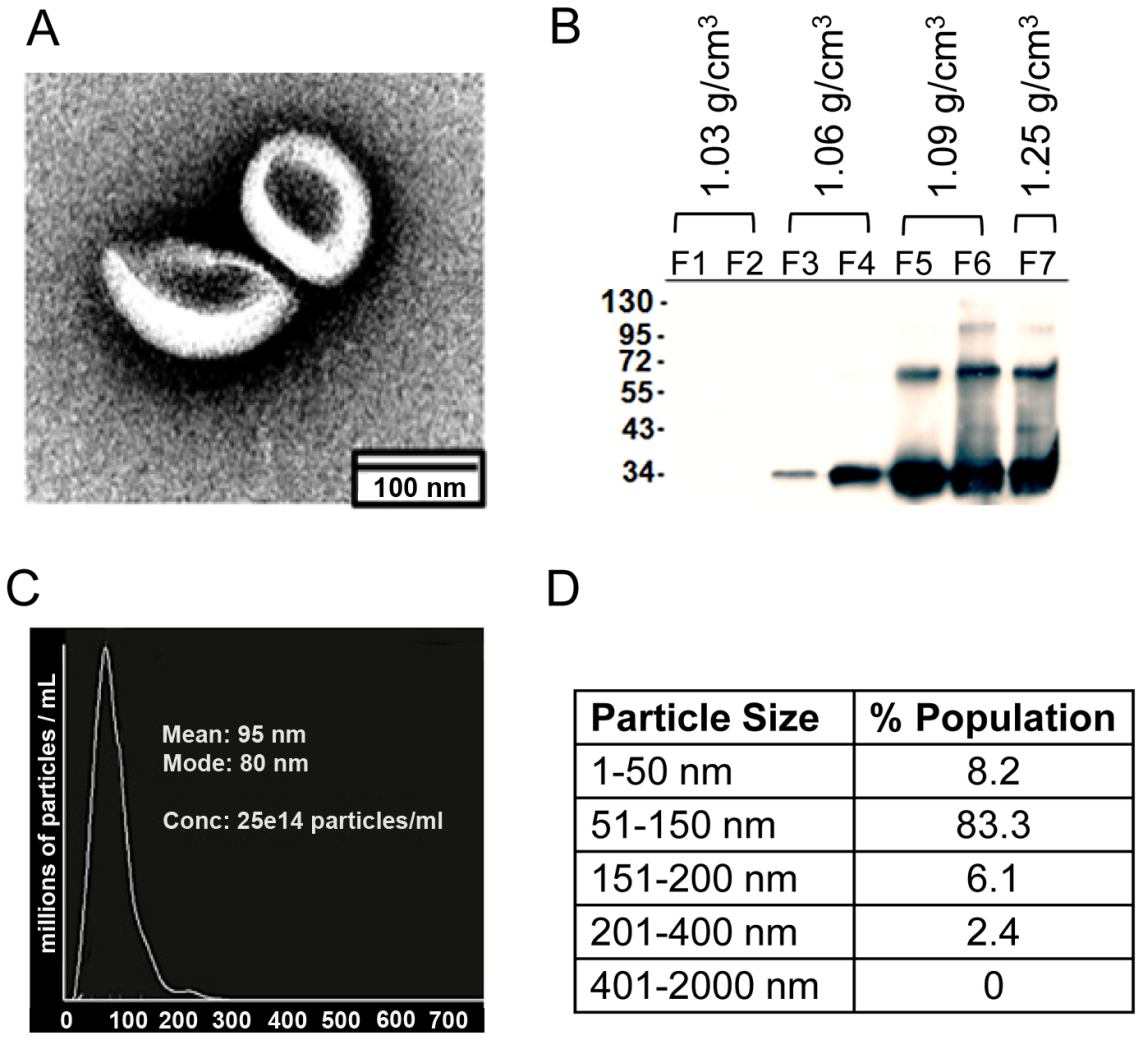
Physical characterization of *T. vaginalis* exosomes. (A) Cup-shaped vesicles ∼50–100 nm in diameter were observed using negative staining EM. (B) Immunoblot analyses of sucrose gradient fractions (F1–F7) containing Tsp1-HA tagged exosomes reacted with anti-HA antibodies. Densities are listed above the fractions and molecular weight markers (in kDa) are shown on the left. Expected size of Tsp1-HA is ∼34 kDa, the band at 72 kDa is likely a dimer. *T.vaginalis* vesicles have a density of ∼1.06–1.25 g/cm^3^. (C) Nanosight trace of purified vesicles. A mean diameter of 95 nm was measured. (D) Table from Nanosight analysis of percentage of purified vesicles in various size ranges.

To test whether *T. vaginalis* produces exosomes, vesicles were isolated from parasite growth media through a series of ultracentrifugation steps, similar to that described for isolating mammalian exosomes [32]. Examination of the preparation by electron microscopy (EM) revealed cup-shaped vesicles of ∼50–100 nm (Fig. 2A), similar in size and shape to mammalian exosomes [33]. To determine if the vesicles had the density reported for mammalian exosomes, vesicles containing a hemagglutinin (HA) tagged Tsp1 (Tsp1-HA) for tracking purposes were floated on a linear sucrose density gradient. The Tsp1-HA-tagged vesicles were found to have densities of 1.03–1.25 g/cm^3^ (Fig. 2B), similar to that reported for mammalian exosomes (∼1.1–1.2 g/cm^3^, [34]). The higher MW band in Fig. 2B is most likely a homodimer of Tsp1 as tetraspanin proteins are known to form dimers [35]. To determine vesicle size variation, we used nanoparticle tracking analysis (Nanosight, Costa Mesa, CA) to directly examine millions of vesicles. This analysis showed that the size of vesicles peak with a mean diameter of 95 nm and 83.3% are between 50–150 nm in size (Fig. 2C and 2D).

**Figure 3:**
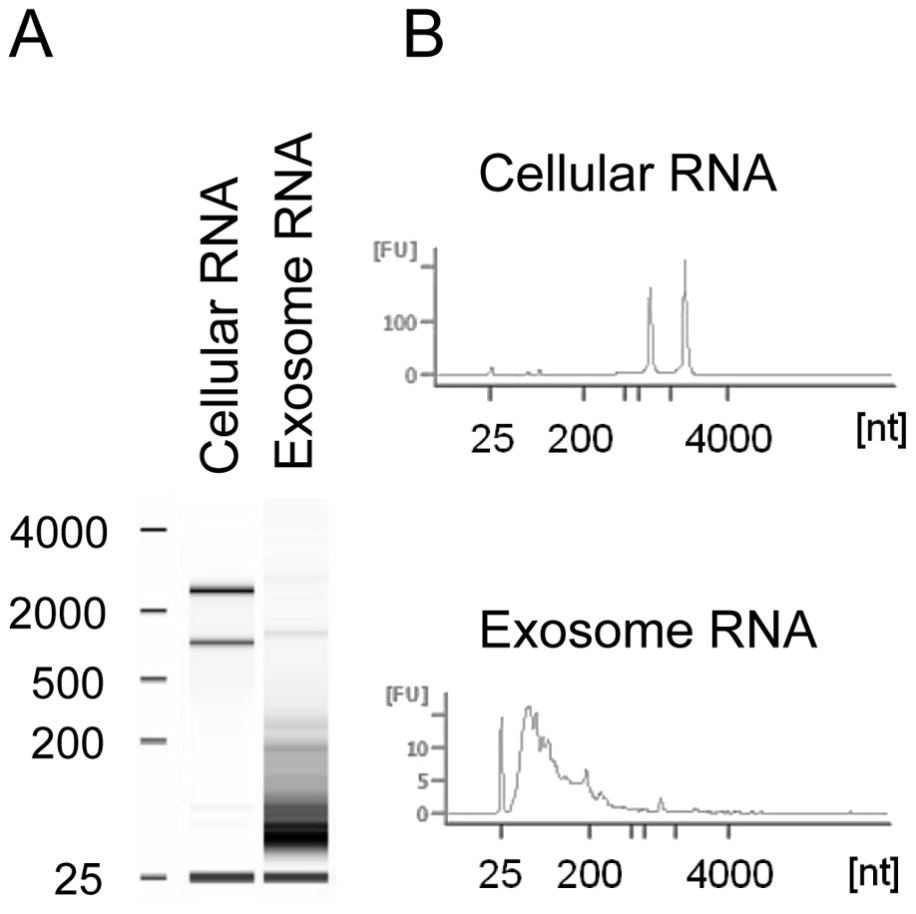
Exosomal RNA. (A) Mock gel of cellular and exosomal RNA analyzed using an Agilent 2100 Bioanalyzer. Molecular weight markers (in nucleotides (nts)) are indicated on the left. (B) Traces with the amounts of cellular or exosomal RNAs relative to size in nts are shown.

We then determined whether these vesicles contain RNA using an Agilent 2000 Bioanalyzer, as mammalian exosomes have been reported to deliver miRNAs and mRNAs to recipient cells [26]. A heterogenous population of small RNAs ranging in size from between 25 and 200 nt were found (Fig. 3A and3B). Taken together, the size, morphology, density, and presence of Tsp1 and RNA indicates that *T. vaginalis* produces and excretes exosomes.

### The *T. vaginalis* exosome proteome

**Figure 4:**
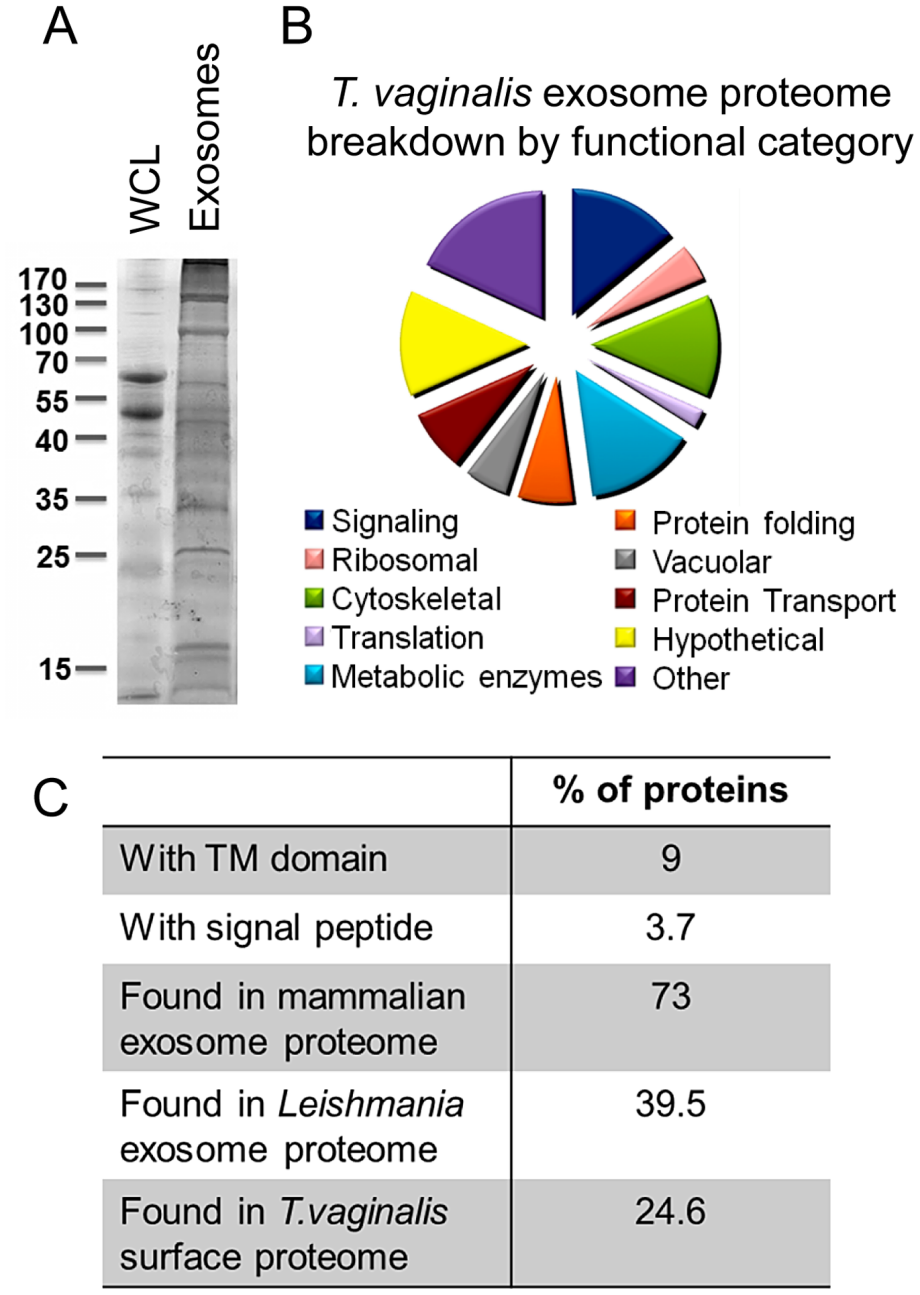
Exosomal Protein Contents. (A) Silver stained polyacrylamide gel of whole cell lysate (WCL) and isolated exosomes, normalized by protein concentration. (B) The predicted function of the 215 proteins identified in exosomes isolated from strain B7RC2. (C) Table of percentage of exosomal proteins with transmembrane domain or signal peptides as annotated in TrichDB (www.trichdb.org) as well as percentage of exosomal proteins with orthologs in mammalian exosomes (ExoCarta database), *Leishmania* exosomes [37], or *T.vaginalis* surface proteome [15].

SDS-PAGE followed by silver staining of proteins of exosomes and whole cell lysates normalized by protein concentration indicates an enrichment of specific proteins in exosomes (Fig. 4A). To define the proteins packaged in adherent *T. vaginalis* B7RC2 strain exosomes and compare their contents with exosomes from other eukaryotes, we determined their protein composition using MudPIT-based proteomic mass spectrometry. Proteins with two or more identified peptides that were found in at least three of seven MudPIT analyses were included in the exosome proteome and revealed a total of 215 proteins (Table S1). These inclusion parameters are conservative compared with other exosomal proteome analyses wherein proteins identified in two of ten experiments or proteins with one peptide in a single experiment were included [36], [37].

When compared with the compiled list of common mammalian exosome proteins in ExoCarta [38], we found that *T. vaginalis* exosomes contained orthologs of approximately 73% of mammalian exosome proteomes and 39.5% are orthologous to *Leishmania* exosomal proteins [39]. The extensive overlap with mammalian exosomal proteins was surprising as roughly 2/3 of *T. vaginalis* genes have no mammalian orthologs [40]. Shared proteins represent 60 core conserved exosome protein/protein families such as tetraspanins, Alix, Rabs, Hsp70, subunits of heterotrimeric G proteins and TcTP [25] as well as hypothetical proteins identified by BLAST analyses as similar to mammalian exosomal proteins. Identified proteins were sorted into functional groups by BLAST analyses and genome annotation and assigned a predicted function (Fig. 4B). Fourteen percent are signaling proteins, 14% are metabolic enzymes, 13% are cytoskeletal proteins, 8% are involved in transport and 6% are vacuolar proteins. The remaining proteins include 32 hypothetical proteins (15% of total) of unknown function as well as proteins involved in protein folding and other cellular activities (Fig. 4B and Table S1). It is notable that one protein in the exosome proteome Tvag_452120 (TvG402) was previously found to localize to in large vesicular structures within *T.vaginalis* [41]. Additionally, 24.6% were previously found in the *T.vaginalis* surface proteome [15]). Nine percent have predicted transmembrane domains and 3.7% have predicted signal peptides as annotated in the TrichDB database (Figure 4C). It should be noted, however, that *T. vaginalis* membrane proteins often appear to lack conventional, identifiable N-terminal signal peptides or transmembrane domains [15], making it difficult to predict whether many exosomal proteins are membrane associated or soluble. Nevertheless, the high degree of conservation between mammalian and *T. vaginalis* exosome proteomes firmly establishes that *T. vaginalis* secretes exosomes.

Novel proteins that may have a role in *T. vaginalis* pathogenesis are also present in the exosome proteome. Interestingly we identified surface proteins (Tvag_240680, BspA family) and proteases (Tvag_224980, metallopeptidase) thought to be involved in pathogenesis [6], [40], [42], [43]. For example, Tvag_340570 is related to two surface proteins involved in parasite attachment which are significantly more abundant in highly adherent versus poorly adherent parasites [15]. These adhesion related surface proteins are part of a ∼150 member family, 30% of which have EST evidence for expression (www.trichdb.org) and 18% of which were found in the surface proteome [15]. Orthologs of virulence proteins characterized in other parasites (Tvag_371800, GP63-like) and those potentially involved in host immune regulation (Tvag_137880, peptidyl proyl isomerase A also known as cyclophilin A) are also notable. Many of the identified exosomal proteins are members of very large gene families [40]; however, only 1 or 2 members are packaged in the exosome, indicating a specificity in expression and/or packaging of specific proteins. For example, there are 911 putative BspA family proteins, ∼30% of which have EST, RT-PCR, or microarray data to support expression [43] and while 11 were found in the surface proteome only 1 was identified in the exosome proteome. Additionally there are ∼700 proteases encoded in the genome, of which ∼120 are annotated as metallopeptidases with 60% having EST evidence for expression and only 3 are found in the exosome proteome. Similarly, ∼90 GP63-like proteases are annotated in the genome, 33% have been shown to be expressed, 16 were found in the surface proteome [15], while only 1 was identified in the exosome proteome. Future studies on the differential expression of proteins packaged into exosomes by *T.vaginalis* strains with varying virulent phenotypes should help identify exosomal proteins involved in pathogenesis..

### *T. vaginalis* exosomes fuse with and deliver proteins to host cells

**Figure 5:**
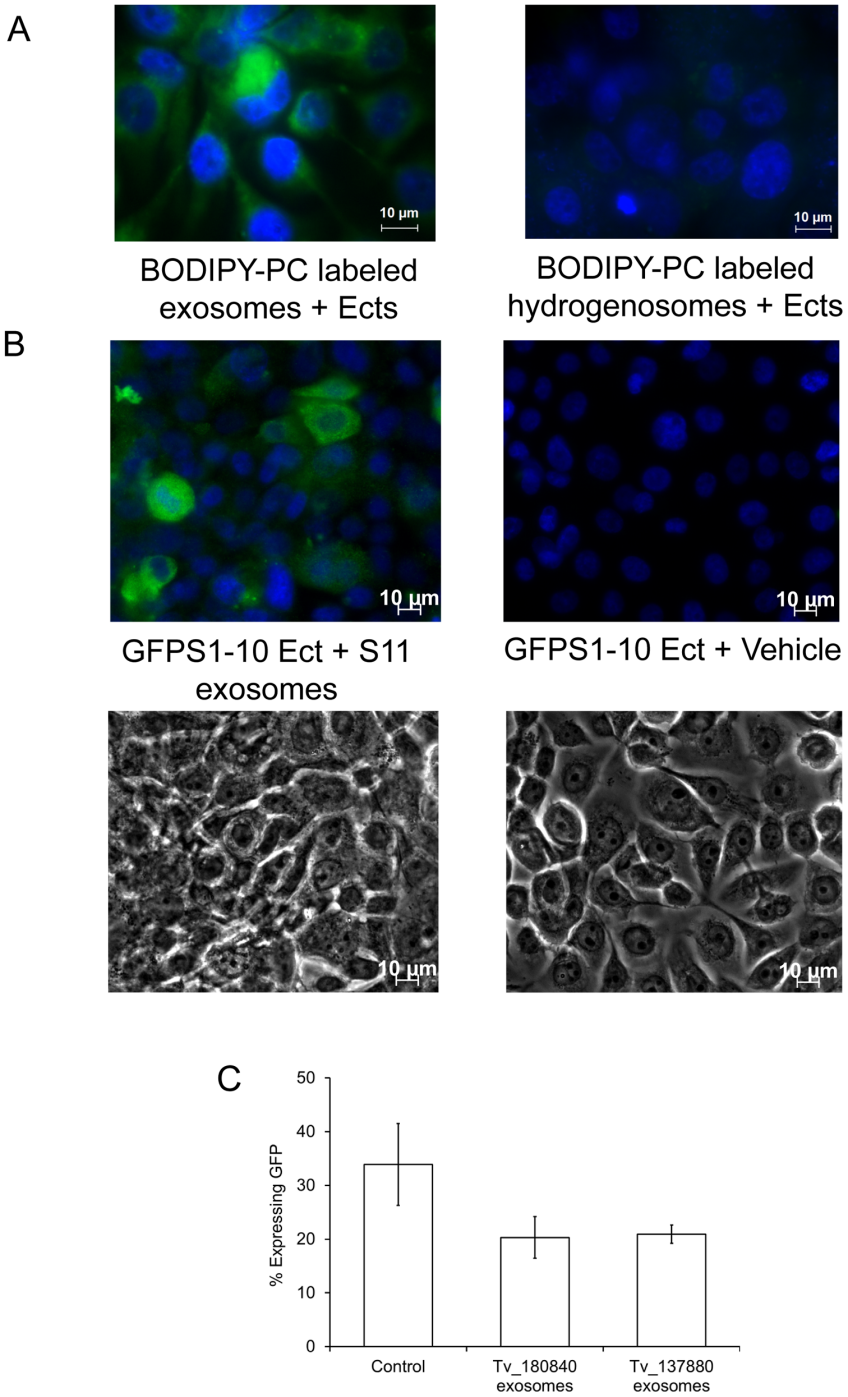
BODIPY-PC labeled exosomal membranes transfer the label to Ects. (A) BODIPY-PC labeled exosomes or BODIPY-PC labeled hydrogenosomes were incubated with Ects. After extensive washing, cells were DAPI-stained & viewed by fluorescence microscopy. (B) Ects transiently transfected with GFPS1-10 were incubated for 1 hr with exosomes from *T. vaginalis* exosomes expressing GFPS11 tagged proteins. Ects were washed, fixed, DAPI-stained & viewed by fluorescent microscopy. Incubation with exosomes containing the Tvag_180840 protein tagged with the GFPS11 fragment is shown; similar data was obtained using a second (Tvag_137880) exosomal protein fused with the GFPS11 fragment. (C) Quantification of GFP fluorescence for a representative experiment, showing mean ± standard deviation. Approximately 35% of Ects were transfected using a control full length GFP (left) whereas ∼20–25% of Ects fluoresce when Ects are transfected with GFPS1-10 and incubated with either Tv_180840 exosomes (middle) or Tv_137880 exosomes (right).

Increasing evidence indicates exosomes are capable of mediating cell∶cell communication, leading to intercellular transfer of molecules [44], [45]. Having established that *T. vaginalis* exosomes contain several proteins potentially involved in pathogenesis, we hypothesized that these exosomes could be used by the parasite during infection. The host cells first encountered by *T. vaginalis* during infection that are the primary site of replication and survival are ectocervical cells (Ects) [46]. We examined whether *T. vaginalis* exosomes associates with Ects by labeling exosomal membranes with BODIPY-PC [47], followed by incubation with Ects. After extensive washing to remove free exosomes, Ects were examined by microscopy and the fluorescent BODIPY-PC was found to label the Ects (Fig. 5A). This is in contrast with that observed using BODIPY-PC labeled hydrogenosomes [48] (Fig. 5A) which are not capable of transferring BODIPY-PC to Ects. These data indicate that *T. vaginalis* exosomes deliver their contents to host cells.

To directly examine whether soluble protein in the parasite exosomes are delivered to host cells we utilized a split-GFP system [49]. Ects were transiently transfected with the large S1-10 fragment of GFP. Exosomes were purified from *T.vaginalis* expressing a soluble exosome protein (Tvag_180840, TcTP or Tvag_137880, peptidyl proyl isomerase A) tagged with the small S11 fragment of GFP. These exosomes were then incubated with GFPS1-10 transfected Ects. GFP fluorescence will only be observed if the S11 fragment fused in frame with an exosomal protein is delivered to the cytoplasm of an Ect containing the S1-10 fragment. As shown in Fig. 5B, fluorescence was observed specifically in exosome-treated and not in vehicle treated Ects. After normalization by transfection efficiency which was ∼30%, quantification of the data estimates that exosomes fused with ∼50% of the Ects (Fig. 5C). These results provide strong evidence that *T. vaginalis* exosomes fuse with and deliver their contents to host cells.

### *T. vaginalis* exosomes are immunomodulatory

**Figure 6:**
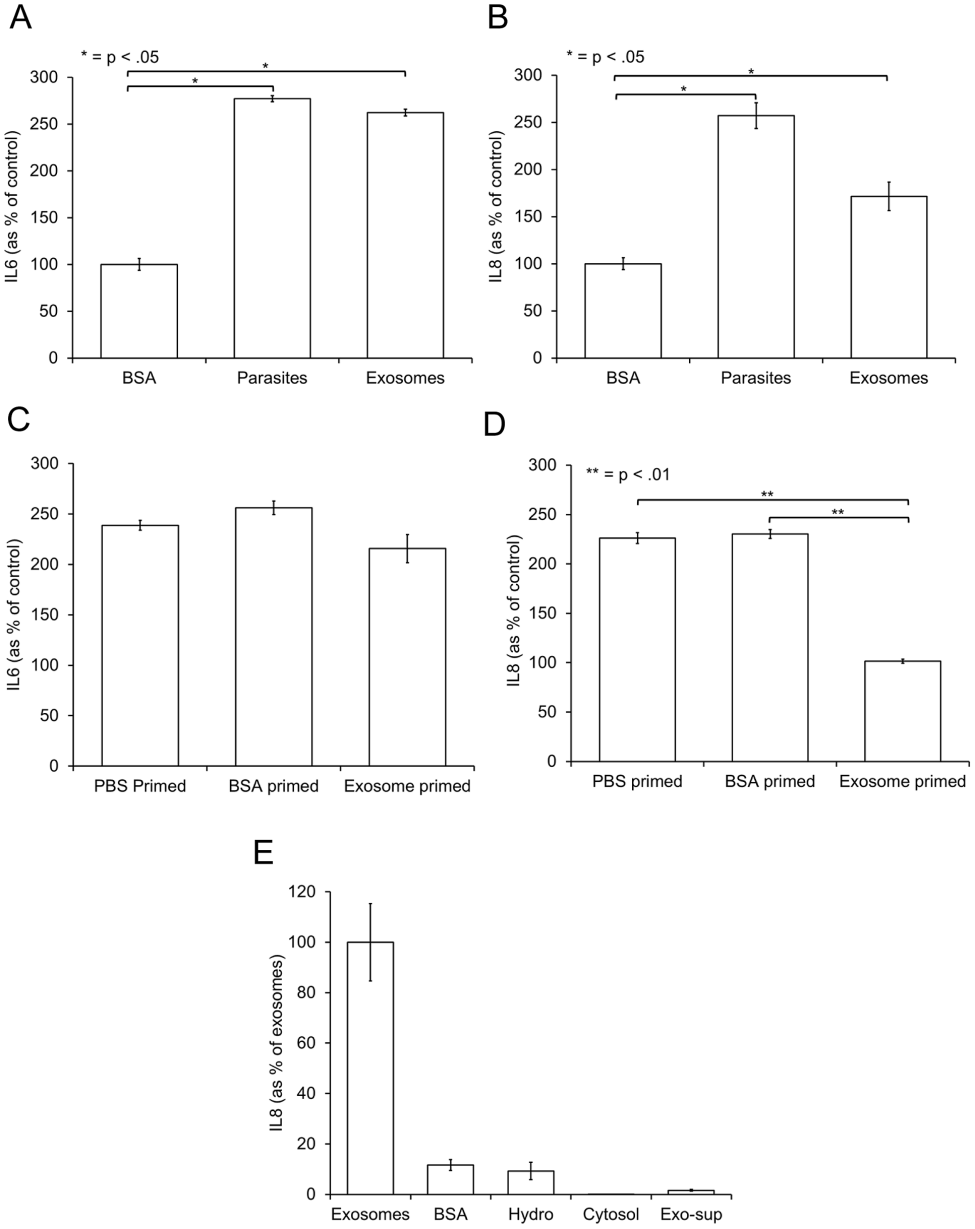
Exosomes induce Ect secretion of IL6 and IL8 and specifically prime the VEC IL8 response to parasites. (A & B) Ects were incubated with either 9 ug BSA, 5×10^5^ parasites, 9 µg exosomes for 6 hr and the supernatants were removed and assayed for IL6 or IL8 cytokines as indicated using ELISA assays. (C & D) Ects were preincubated for 12 hr with either PBS, 9 ug BSA, or 9 ug exosomes followed by the addition of 5×10^5^ parasites for 6 hr. Supernatants were then assayed for either IL6 or IL8 cytokines as indicated and data were normalized as percent of cytokine secretion using 9 ug BSA without the addition of parasites. (E) Ects were incubated with 9 ug of either exosomes, BSA, hydrogenosomes, cytosol or supernatant from the last step of the exosome purification protocol, as indicated, for 6 hr. Supernatants were spun to remove debris and then assayed for IL8. Data was normalized as percent of IL8 secretion using 9 ug exosomes as 100%. Mean values ± standard deviation of a representative experiment is shown.

We hypothesized that *T. vaginalis* exosomes may modulate the Ect immune response, as these cells are involved in host innate immunity [12], [50]–[53]. Specifically, we examined the proinflammatory cytokines IL6 and IL8 secreted by Ects in response to both exosomes and parasites. To allow comparison, we used the concentration of exosomes predicted to be produced by the number of parasites used in the same experiment as calculated from exosome isolation yields. Quantification of IL6 secretion by Ects demonstrated that *T. vaginalis* exosomes induce this cytokine to approximately the same extent as parasites (Fig. 6A). Exosomes were also shown to elicit IL8 secretion; however, the response is only ∼50% of that observed when Ects are exposed to parasites (Fig. 6B). It is notable that using equivalent amount (9 ug) of either *T. vaginalis* hydrogenosomes, cytosol or exosomal supernatant did not induce a cytokine response (Fig. 6E). Because IL6 is an acute inflammation protein and IL8 is involved in a long-term inflammatory process [54] we further hypothesized that the exosomes secreted by *T. vaginalis* could potentially prime host cells for parasite infection. We found pretreating Ects with exosomes did not affect their subsequent IL6 cytokine production (Fig. 6C). However, as shown in Fig. 6D, preincubation of Ects with exosomes prior to the addition of *T.vaginalis* parasites led to a significant inhibition of IL8 secretion by the Ects. These results indicate that *T. vaginalis* exosomes specifically modulate IL8, but not IL6 production by Ects.

### *T. vaginalis* exosomes mediate parasite∶parsite interaction and are involved in adherence to host cells

**Figure 7:**
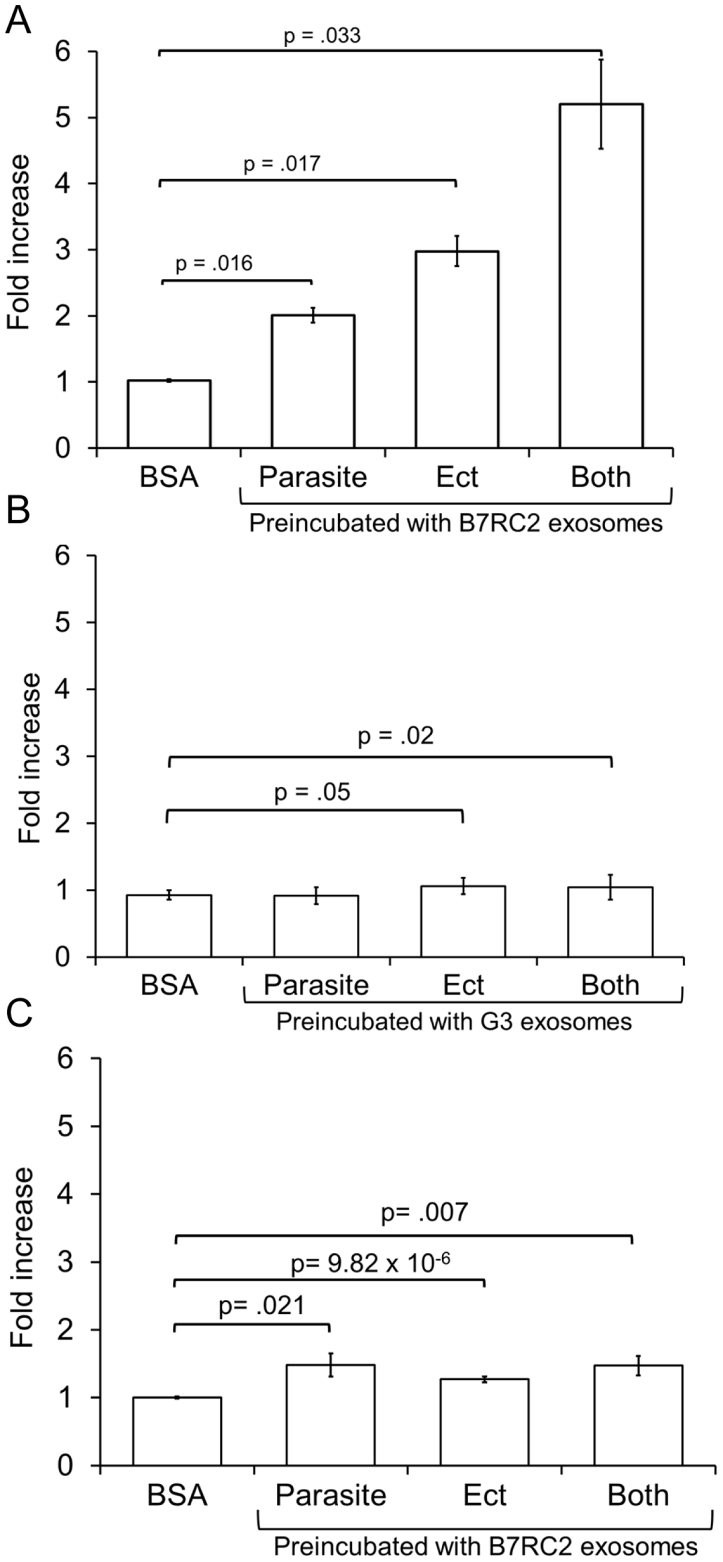
Preincubation with exosomes of a highly adherent strain increases adherence of a poorly adherent strain to Ects. Poorly adherent parasite strain G3, Ects or both, as indicated, were preincubated with exosomes from the highly adherent B7RC2 strain (A) or poorly adherent G3 strain (B) for 1 hr, followed by washing to remove exosomes. Adherence of G3 parasites to the Ects was then measured. BSA preincubation of Ects (indicated as BSA) served as a negative control and was used to normalize experiments. (C) B7RC2 (highly adherent) parasites, Ects, or both, as indicated, were preincubated with exosomes from B7RC2 for 1 hr, followed by washing, and determination of adherence. The mean of three experiments done in triplicate per experiment is shown ± SEM.

Secreted exosomes can potentially interact with other parasites in the population such that exosomes from one parasite might influence another. As attachment to host cells is critical for establishing infection [6], [7], we examined whether exosomes purified from B7RC2, a strain 20-fold more adherent than the lab strain G3 [15], affects the attachment of the less adherent strain G3 to Ects. The ability of exosomes to mediate both parasite∶parasite and parasite∶host cell adherence was assessed. The following were preincubated with B7RC2 exosomes for 1 hour prior to performing attachment assays: 1) G3 parasites only 2) Ects only or 3) both Ects and G3 parasites. Unincorporated exosomes were washed away before performing the attachment assay and as a negative control BSA was used instead of exosomes. Based on average yields of exosomes per parasite number, the amount of exosomes oredicted to be secreted by the quantity of parasites used in our assays was used for all preincubation treatments. Preincubation of B7RC2 exosomes with G3 parasites resulted in a 2-fold increase in G3 attachment to Ects showing that exosomes from one parasite can affect attachment of another parasite (Fig. 7A). Preincubation of B7RC2 exosomes with Ects resulted in a 3-fold increase in G3 attachment to Ects showing that exosomes alter the host cell and result in increased parasite attachment. Interestingly, this effect is additive as a fivefold increase in G3 attachment was observed when both the G3 parasites and Ects are preincubated with B7RC2 exosomes (Fig. 7A). As losses result during isolation of exosomes, we believe the amount of exosomes utilized in our experiments is conservative, however a dose curve (Fig. S2) shows that increasing amounts of B7RC2 exosomes does increase G3 adherence. Contrary to that observed using B7RC2 exosomes, preincubation of parasites, Ects, or both, with exosomes from the poorly-adherent G3 strain did not increase G3 attachment to Ects (Fig. 7B). Furthermore preincubation of B7RC2 exosomes on B7RC2 parasites only had a slight effect on attachment to Ects (Fig. 7C). This is likely due to a saturation effect as the B7RC2 parasites produce and secrete their own exosomes. Doing the identical experiment except replacing exosomes with equivalent amounts of *T. vaginalis* hydrogenosomes or cytosol does not result in increased parasite attachment (Fig. S1A and Fig. S1B, respectively). These data indicate that exosomes from a highly adherent strain can increase parasite attachment of a less adherent strain to host cells. Moreover, they indicate that exosomes can mediate both intraspecies and interspecies interactions.

**Figure 8:**
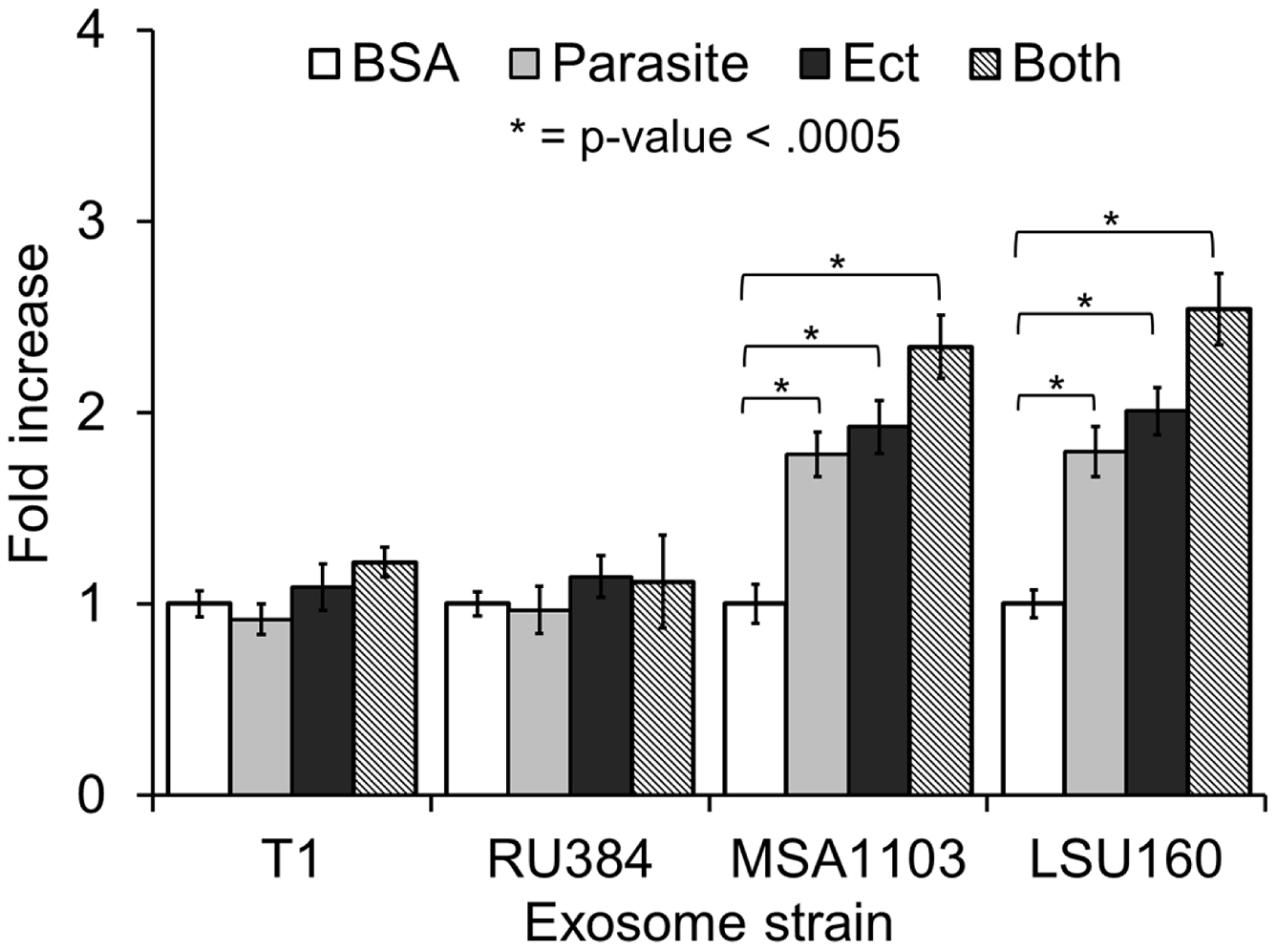
Exosomes from highly adherent strains increase attachment of poorly adherent G3 parasites to host cells while exosomes from strains of equal or lesser adherence do not affect G3 attachment. Poorly adherent G3 parasites, Ects or both, as indicated, were preincubated with exosomes from the T1, RU384, MSA1103, or LSU160 strain for 1 hr, followed by washing to remove exosomes. Adherence of G3 parasites to the VECs was then determined. The mean of 3–5 experiments with each treatment done in triplicate per experiment is shown ± SEM.

**Figure 9:**
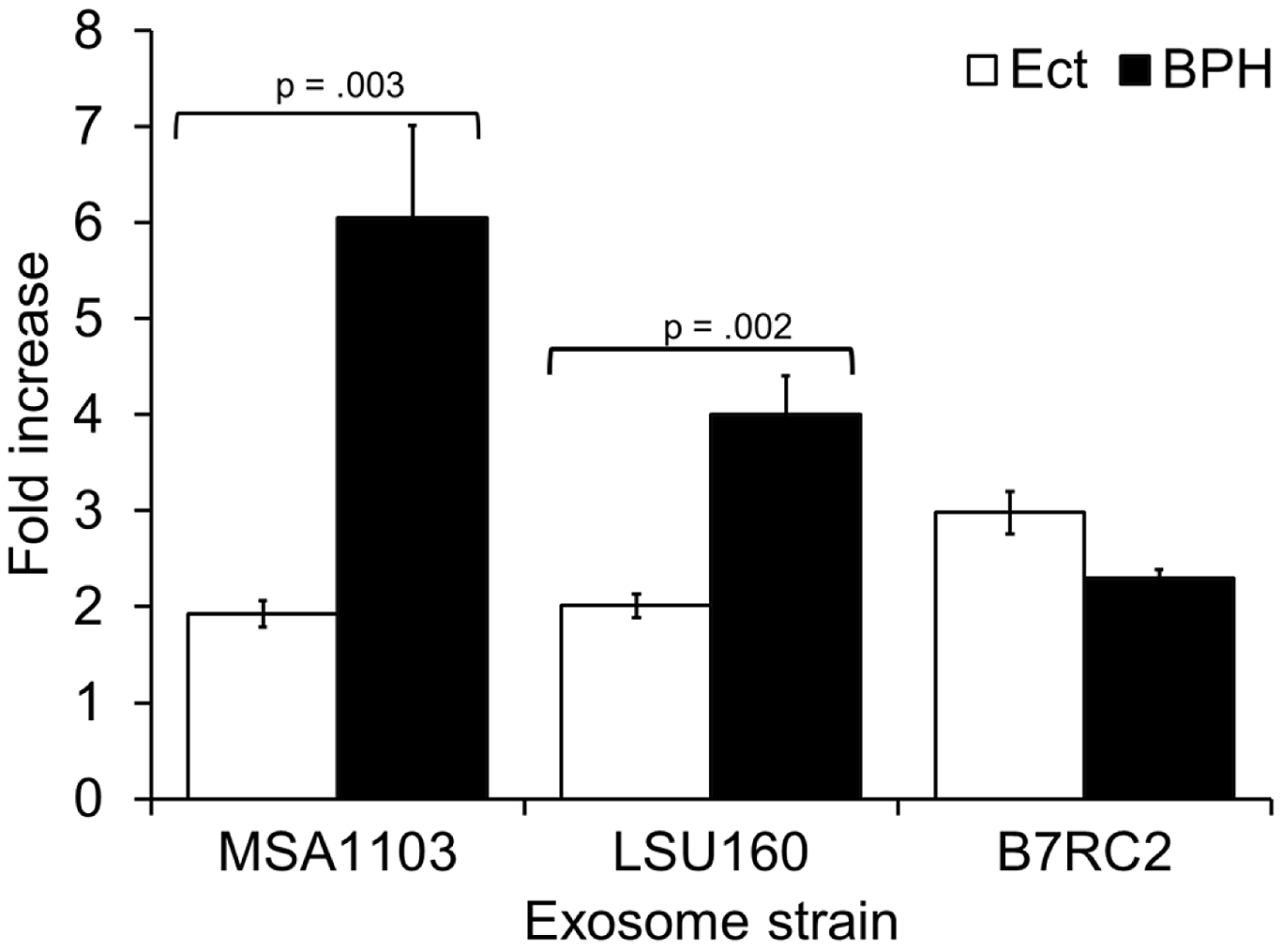
Exosomes from strains more highly adherent to BPH1 cells than Ects have a greater effect on G3 attachment to BPH than to Ects. Ect or BPH1 cells were preincubated with exosomes from either the G3, MSA1103, LSU160, or B7RC2 strain for 1 hr as indicated, followed by washing to remove exosomes. Adherence of G3 parasites to either BPH1 or Ects was measured. BSA preincubation of Ect or BPH1 cells was used to normalize the data. Mean of fold increase in attachment relative to BSA control of 3–5 experiments +/− SEM is shown.

To test whether the parasite∶parasite and parasite∶host interactions observed using the B7RC2 strain is generally observed with other parasite strains, we expanded our G3 attachment assay to include exosomes purified from several strains. We found that exosomes from poorly adherent strains like T1 or RU384 [55] do not significantly increase adherence of G3 parasites while those from more highly adherent strains like MSA1103 or LSU160 [55] do increase attachment of G3 to Ects (Fig. 8). As MSA1103 and LSU160 strains are more adherent (3 and 2 fold respectively) to the benign prostate epithelium cell line (BPH1) than the female Ect host cells [55], we tested whether exosomes from these two strains result in a greater increase in G3 parasite adherence to BPH1 cells as compared to Ects. We found that while exosomes from LSU160 and MSA1103 increased attachment to Ects ∼2 fold, they increased attachment to BPH1 cells by ∼6 and ∼4 fold respectively (Fig. 9). In contrast a difference was not observed when preincubating with B7RC2 exosomes consistent with B7BC2 parasites displaying no preference for attachment to Ects versus BPH1 [55]. Together, these results indicate that exosomes package pathogenic factors specific to the strain that produces them and that exosomes from highly adherent strains may contribute to the parasites' ability to colonize specific niches of the male and female urogenital tract.

## Discussion

This study, the first to identify and characterize exosomes from the parasite *T. vaginalis*, reveals a role for these secreted vesicles in host∶parasite interactions. We have isolated *T. vaginalis* exosomes from the adherent B7RC2 strain and shown that they are remarkably similar to mammalian exosomes in size, structure and core protein components [22], [25], [56]. The extensive (73%) overlap between the *T. vaginalis* and mammalian exosomal proteomes demonstrate the specific packaging of proteins within exosomes as the majority of *T. vaginalis* proteins have no orthologs in humans [40]. Minimal overlap between the *T. vaginalis* exosomal proteome and other reported *T. vaginalis* proteomes [7], [15], [57] and the presence of only 1–2 proteins from large, expressed protein families [6], [40] likewise argues for specificity in exosomal protein content. Exosome biogenesis and the selective packaging of exosomal proteins is poorly understood [20], [58], [59]; however, the overlap of the exosome proteomes of this highly divergent parasite and that of humans indicates the conservation of underlying mechanisms. Thus the ease of culturing *T. vaginalis* and its high exosomes yield makes this parasite a good model for studying the basic biological properties of exosomes.

In addition to the core proteins conserved between *T. vaginalis* and mammalian exosomes, many proteins unique to *T. vaginalis* exosomes were also identified. Thirty-two are conserved hypothetical proteins of unknown function. Others, including several surface proteins and proteases, have been implicated in pathogenesis. Future experiments characterizing the exosomal contents from strains of parasites of varying virulence will assist in identifying and characterizing specific exosomeal proteins that affect pathogenesis. Unique *T. vaginalis* exosomal proteins may be critical for mediating host∶parasite interactions In this regard, another parallel can be drawn between *T. vaginalis* and mammalian exosomes as the latter are involved in interactions that lead to pathologies such as cancer proliferation and HIV transmission between different cell types [28], [60]–[63].

Using a split-GFP assay, parasite exosomes were shown to fuse with and deliver their contents to host cells. *T. vaginalis* exosomes were also shown to modulate host cell cytokine production. *T. vaginalis* may use exosomes to manipulate host defense responses similar to the secretion of virulence factors and vesicles by bacteria [22], [64], [65]. Exosomes were found to induce an IL6 response in Ects and to down regulate the IL8 response to parasites. IL8 is involved in the recruitment of neutrophils to the site of infection and persists in its active form within the immediate environment for longer than other chemoattractants [12], [66]. Thus by dampening the IL8 response of Ects to parasites, *T. vaginalis* exosomes may play a critical role in establishing a successful chronic infection. IL6 is a chief stimulator of proteins in acute inflammation and suppresses the level of other proinflammatory cytokines in an acute response [67]. IL6 can also stimulate IL-1 receptor antagonist, an anti-inflammatory mediator, to control tissue inflammatory responses [68]. Thus *T. vaginalis* exosomes may lead to the regulation of IL6 and IL8 secretion, thus priming the urogenital tract for parasite colonization. Both proinflammatory and immunosuppressive responses to *T. vaginalis* infection or its double-stranded RNA virus have been described in various *in vitro* and mouse studies [69], [70]. Future studies aimed at investigating the effects of exosomes on various host effector cells recruited during infection will be necessary to understand the molecular mechanisms by which exosomes modulate host immune responses.

*T. vaginalis* exosomes were found to substantially increase the adherence of this sexually-transmitted parasite to female (Ect) and male (BPH1) epithelial cells via an effect on both parasites and host cells. As an extracellular parasite, attachment of *T. vaginalis* to host cells is vital for survival and pathogenesis. Similar to that described for exosomes produced by different mammalian tissues and cells [23], [56], [71], *T. vaginalis* exosomes have strain-specific characteristics. Exosomes from highly adherent parasites are capable of increasing the adherence of poorly adhering parasite to host cells, whereas those from poorly adhering parasites are not. Furthermore, exosomes from parasite strains that preferentially bind BPH1 cells are more effective in increasing adherence to BPH1 versus Ects. Taken together, our data indicate *T. vaginalis* exosomes may package strain specific, perhaps even host cell specific, virulence factors. Future studies on differential packaging of factors in exosomes between strains may lend insight into differences attributed to virulent and less virulent phenotypes.

*T. vaginalis* exosomes could potentially be found in vaginal secretions or urine of infected individuals and thus serve as biomarkers for infection. Testing for infection in females currently requires gynecology visits [2], [72] and there is a lack of a non-invasive, quick method for diagnosing *T. vaginalis* infection in men [73]. Exosomes have been successfully isolated from a variety of easily obtained bodily fluids including blood, amniotic fluid, saliva, and urine [74]. It has been shown that urine contains abundant amounts of exosomes from prostatic secretions [75] and hence could contain exosomes from *T.vaginalis* in infected patients. Thus the isolation of *T. vaginalis* exosomes or detection of exosomal contents may provide a non-invasive means to diagnose infection. Furthermore, the ability of *T. vaginalis* exosomes to deliver soluble substances to host cells provides the potential for parasite exosomes to be used therapeutically.

Mammalian exosomes and their RNA and protein contents have been shown to regulate a variety of cellular pathways by modulating gene expression in recipient cells [76], [77]. The characterization *of T. vaginalis* exosomes and their protein cargo sets the stage for determining specific parasite factors likely to modulate host cell response and affect infection outcomes. The small RNAs packaged inside *T. vaginalis* exosomes may also modulate parasite∶parasite or parasite∶host interactions. The possibility that these small RNAs are novel parasite miRNAs that are delivered to the host cell to modulate gene activity is an appealing idea.

**Figure 10:**
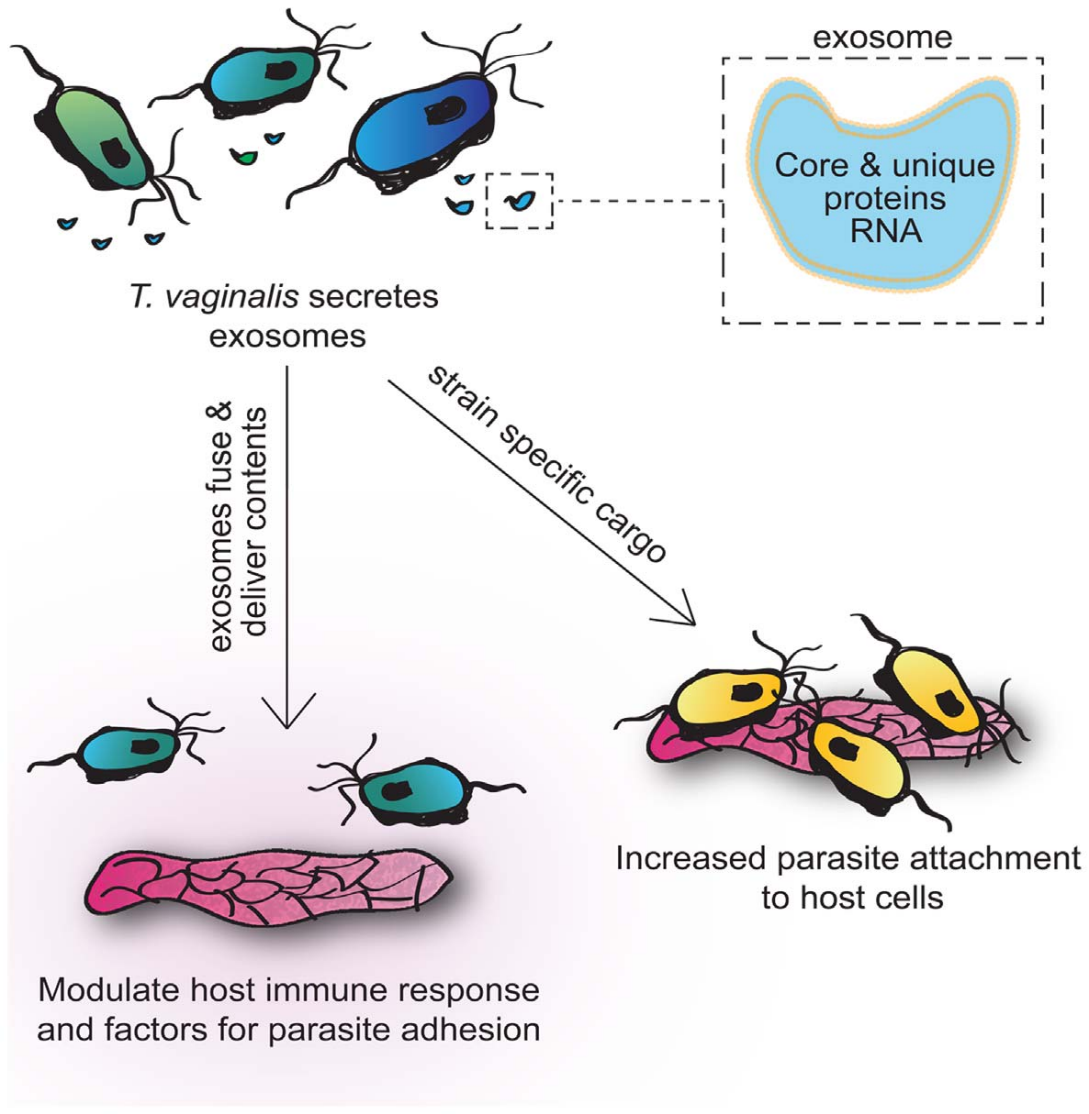
Visual summary of role of *T.vaginalis* exosomes. *T.vaginalis* secretes exosomes that package core conserved exosomal proteins as well as those unique to the parasite that may be involved in pathogenesis. Contents of exosomes may be strain specific and vary in their ability to increase parasite attachment to host cells. Parasite exosomes can fuse with hose cells and deliver their contents. These contents modulate host immune response and potentially factors for parasite adhesion.

The use of exosomes by a strictly extracellular parasite represents a novel method by which the parasite may deliver proteins and/or RNA to the host to manipulate host cell responses while remaining extracellular. The delivery of strain specific exosomal contents can impact both host immune response as well as parasite attachment to host cells (Fig. 10). A better understanding of how exosomes increase parasite adherence to host cells and modulate host cell responses will provide insights into pathogenesis and possibly new avenues for diagnosis and therapy.

## Materials and Methods

### Cell culture

*T. vaginalis* strains (B7RC2, G3, T1, RU384, MSA1103, LSU160) were cultured in TYM medium supplemented with 10% horse serum, 10 U/ml penicillin/10 ug/ml streptomycin (Invitrogen), 180 uM ferrous ammonium sulphate and 28 uM sulfosalicylic acid [78]. 100 ug/mL G418 (Invitrogen) was added to culture of the Tsp1-HA (Tvag_019180), Tvag_180840, and Tvag_137880 transfectants. Parasites were grown at 37°C and passaged daily for ≤2 weeks. The human cervical epithelial cell line Ect1 E6/E7 (ATCC CRL-2614) was grown as described [46] except without additional CaCl_2_. The human benign prostate epithelial line BPH1 [79] was grown as described [80].

### Exosome isolation

*T.vaginalis* at a density of ∼1.0×10^6^ parasites/ml, were washed 3X and resuspended in TYM media without serum for 4 hrs after which parasites were removed by centrifugation at 500×g. The cell-free media was filtered through 0.22 um filter and concentrated using a Vivaflow 200 100,000 MWCO PES (Sartorious Stedium). Exosomes were pelleted by ultracentrifugation at 100,000×g for 75 min using a TLA100 rotor followed by resuspension in 2 mL cold PBS+1X HALT protease inhibitors (Thermo Scientific). Exosomes were concentrated further by ultracentrifugation at 100,000×g for 70 min and resuspended in 100–300 uL PBS to be purified by floatation on a linear sucrose gradient as described (Raposo, 1996). Pellets were resuspended by boiling for 5 min in Laemmli sample buffer and stored at −20°C in PBS or immediately resolved by SDS-PAGE followed by western blot analysis or silver staining using standard procedures. Fractions transferred to PVDF membranes were blocked with 5% TBST-milk and probed with an anti-HA antibody (1∶5000, Covance) followed by reaction with anti-mouse-HRP (Jackson Labs). Exosome protein concentration was assayed using the Pierce BCA Kit (Thermo Scientific).

### Electron microscopy

Freshly isolated exosomes were directly adsorbed onto charged carbon-coated grids, contrasted with 1% uranyl acetate, and examined using a transmission electron microscope.

### RNA isolation and analysis

Total RNA was isolated from freshly isolated exosomes or whole parasites using the MirVana Paris kit (Invitrogen) according to manufacturer's protocol. The eukaryote total RNA Nano assay (RNA 6000 Nano kit, Agilent) was used to analyze the RNA on an Agilent 2100 Bioanalyzer at the UCLA Clinical Microarray Core.

### Immunolocalization

Parasites were incubated with Ects for 15 or 60 min. Cells were fixed in 4% formaldehyde for 20 min, permeabilized with 0.2% Triton X-100 in PBS, blocked with 3% BSA in PBS (PBS-BSA), incubated with a 1∶1,000 dilution of anti-HA primary antibody (Covance), washed, and then incubated with a 1∶5000 dilution of Alexa Fluor-conjugated secondary antibody (Molecular Probes). The coverslips were mounted using ProLong Gold Antifade reagent with DAPI (Invitrogen). Stained parasites were examined using an Axioskop 2 epifluorescence microscope (Zeiss), and images were recorded with an AxioCam camera and processed with the AxioVision 3.2 program (Zeiss).

### Secretion assay

Parasites at log growth were resuspended in 5% PBS-sucrose at a density of 1×10^6^ parasites/mL at 16°C or 37°C for 1 hour. Parasites were pelleted by centrifugation and the supernatant was filtered through 0.22 um filter to remove cell debris and concentrated using an Amicon filter. The pellet was resuspened to the same volume as the filtered supernatant (700 uL), and 15 uL of each was subjected to SDS-PAGE. Western blot analysis was performed using anti-HA (1∶5000; Covance) to detect Tsp1, anti-β-tubulin (1∶1000; Sigma) and anti-neomycin phosphotransferase II (1∶2500; Jackson Labs). Secondary antibodies were anti-mouse-HRP (1∶25000) and anti-rabbit-HRP (1∶25000).

### MudPIT mass spectrometry

Exosomes were fractionated on 12% Tris-glycine gels (Invitrogen) followed by fixation with 10% methanol and 7% acetic acid. Gel slices were excised and treated with 100 mM ammonium bicarbonate (Fisher) and 50% acetonitrile (ACN). Disulphide bonds were reduced with 10 mM DTT and SH groups alkylated with 50 mM iodoacetamide (Sigma). After washing, gel pieces were dehydrated with ACN then rehydrated on ice with 20 ng/uL trypsin, 40 mM ammonium bicarbonate, 9% ACN, and incubated overnight at 37°C. Acidic peptides were extracted by the addition of 100 mM ammonium bicarbonate and extraction of basic peptides was performed with 2.5% trifluoroacetic acid (TFA). The supernatants were combined, dried in a speed-vac. The dried samples were resuspended in digestion buffer (100 mM Tris-HCl, pH 8, 8M urea), proteolytically digested by the sequential addition of Lys-C and trypsin proteases and subjected to MudPIT analyses as described [15].

### BODIPY-PC membrane transfer assay

10 uM BODIPY-PC (2-decanoyl-1-(O-(11-(4,4-difluoro-5,7-dimethyl-4-bora-3a,4a-diaza-s-indacene-3-propionyl)amino)undecyl)-sn-glycero-3-phosphocholine; Invitrogen) was used to label exosomal or hydrogenosomal membranes for 30 min at 4°C in the dark. Excess lipids were removed by two 500X volume washes with PBS and ultracentrifugation at 50,000 rpm for 1 hr. BODIPY-PC labeled exosomes or hydrogenosomes were added to Ects for 24 hr after which cells were washed with warm media 3X followed by fixation with 4% formaldehyde and mounting with ProLong Antifade reagent with DAPI (Invitrogen). Cells were visualized as described for immunolocalization.

### Split GFP analyses

The pCMV-mGFP-S1-10 mammalian optimized plasmid (Theranostech, Inc) was transiently transfected into Ect cells using Fugene HD (Promega) according to manufacturer's protocol. Tvag_137880 and Tvag_180840 were cloned into Master-Neo plasmid with S11 fused in frame using PCR and parasites were transfected and cultured as described [81]. Exosomes containing S11-tagged proteins were added 48 hr post transfection, allowed to interact for 4 hr and cultures were then washed 3X to remove unfused exosomes. Cells were fixed with 4% formaldehyde and mounted with ProLong Gold antifade reagent with DAPI (Invitrogen) and viewed as described for immunolocalization.

### Measurement of IL6 and IL8 secretion by host cells

Ects were plated in 48-well plates and grown to confluence. 5×10^5^ parasites or 9 ug exosomes were added for 6 hr and the supernatant was then removed. Cells/debris were removed by spinning at 5000 rpm for 10 min. Supernatants were stored at −20°C. IL6 and IL8 was quantified using IL6 or IL8 ELISA kits from AssayPro following manufacturer's directions. For priming experiments, exosomes, BSA, or PBS was added to host cells for 12 hr after which host cells were rinsed 3X with prewarmed KSFM (Invitrogen) and 5×10^5^ parasites were added for 6 hr. Data were normalized as percent of cytokine secretion using 9 ug BSA without the addition of parasites.

### Attachment assay

Attachment of parasites to Ect or BPH1 cells was performed as described [15]. Briefly, CellTracker Blue CMAC (Invitrogen) labeled parasites were added to confluent monolayer of host cells (1∶3 parasite∶host cell ratio) for 30 min. Coverslips were subsequently rinsed in PBS to remove unattached parasites, fixed with 4% formaldehyde (Polysciences, Inc), and mounted on slides with Mowiol (Calbiochem).Fifteen 10X magnification fields were analyzed per coverslip with three coverslips per treatment per experiment. Fluorescent parasites adhered to host cells were quantified using ImageJ.. When examining the role of exosomes in attachment, exosomes were preincubated with Ects, parasites, or both for 1 hr prior to attachment followed by washing with warm media to remove remaining exosomes. All experiments were performed 3–5 times with 3 coverslips per treatment per experiment. Fold change in parasite number was calculated by totaling the number of parasites for 15 images/coverslip, and averaging all coverslips per treatment condition and dividing by the same number derived using negative control BSA samples.

### Statistical analyses

Graphs were made and statistical analyses performed using Microsoft Excel 2010. Independent experiments were performed a minimum of 3 times with at least three technical replicates per experiment. Two sample t-tests were used to determine significance. Data are expressed as standard error of the mean (± SEM).

## Supporting Information

Figure S1 **Parasite cytosol or hydrogenosomes do not increase parasite attachment to Ects.** Poorly adherent parasite strain G3, Ects or both, as indicated, were preincubated with purified cytosol (A) from the highly adherent B7RC2 strain or hydrogenosomes (B) for 1 hr, followed by washing. Adherence of G3 parasites to the Ects was then measured. BSA preincubation of Ects (indicated as BSA) served as a negative control and was used to normalize experiments. The mean of three independent experiments each done in triplicate is shown ± SEM.
(TIF)

Figure S2 **Exosomes increase parasite attachment to Ects in a dose dependent manner.** Poorly adherent parasite strain G3 (A), Ects (B) or both (C), were preincubated with 0, 4.5, 9, or 18 ug of exosomes from the highly adherent B7RC2 strain, followed by washing to remove exosomes. Adherence of G3 parasites to the Ects was then measured. BSA preincubation of Ects (indicated as BSA) served as a negative control and was used to normalize experiments. The mean of three independent experiments each done in triplicate is shown ± SEM.
(TIF)

Table S1 **Characterization of the proteins identified in the** ***T. vaginalis*** **(strain B7RC2) exosomal proteome.** The TrichDB ID# (www.trichdb.org) and the functional category of the protein are shown. The spectral count, an estimate of a peptide's relative abundance (Count) and the % coverage of the protein (Coverage) are listed. The annonation in TrichDB (Description), the presence of a predicted signal peptide (SP) or transmembrane domain (TM) and the number of paralogous genes in the *T. vaginalis* genome are indicated. An X specifies the presence of an orthogous protein in the exosomes of mammals or *Leishmania* or in the *T. vaginalis* surface proteome.
(XLSX)
