# Supplementary figures and images for: Trichomonas vaginalis Exosomes Deliver Cargo to Host Cells and Mediate Host∶Parasite Interactions
Source: PLoS Pathog. 2013 Jul 11;9(7):e1003482. doi: 10.1371/journal.ppat.1003482 (PMC3708881; doi:10.1371/journal.ppat.1003482)

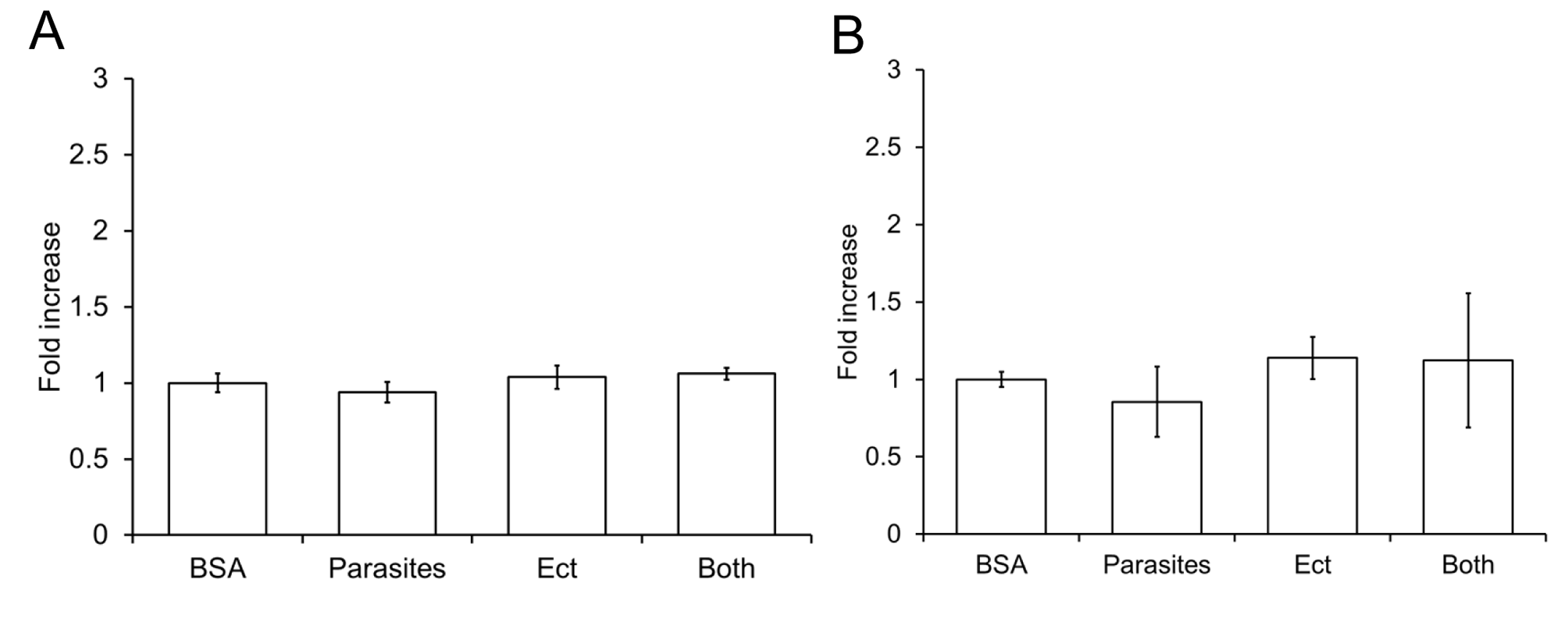

Supplement: Figure S1 — Parasite cytosol or hydrogenosomes do not increase parasite attachment to Ects. Poorly adherent parasite strain G3, Ects or both, as indicated, were preincubated with purified cytosol (A) from the highly adherent B7RC2 strain or hydrogenosomes (B) for 1 hr, followed by washing. Adherence of G3 parasites to the Ects was then measured. BSA preincubation of Ects (indicated as BSA) served as a negative control and was used to normalize experiments. The mean of three independent experiments each done in triplicate is shown ± SEM. (TIF) [file ppat.1003482.s001.tif]

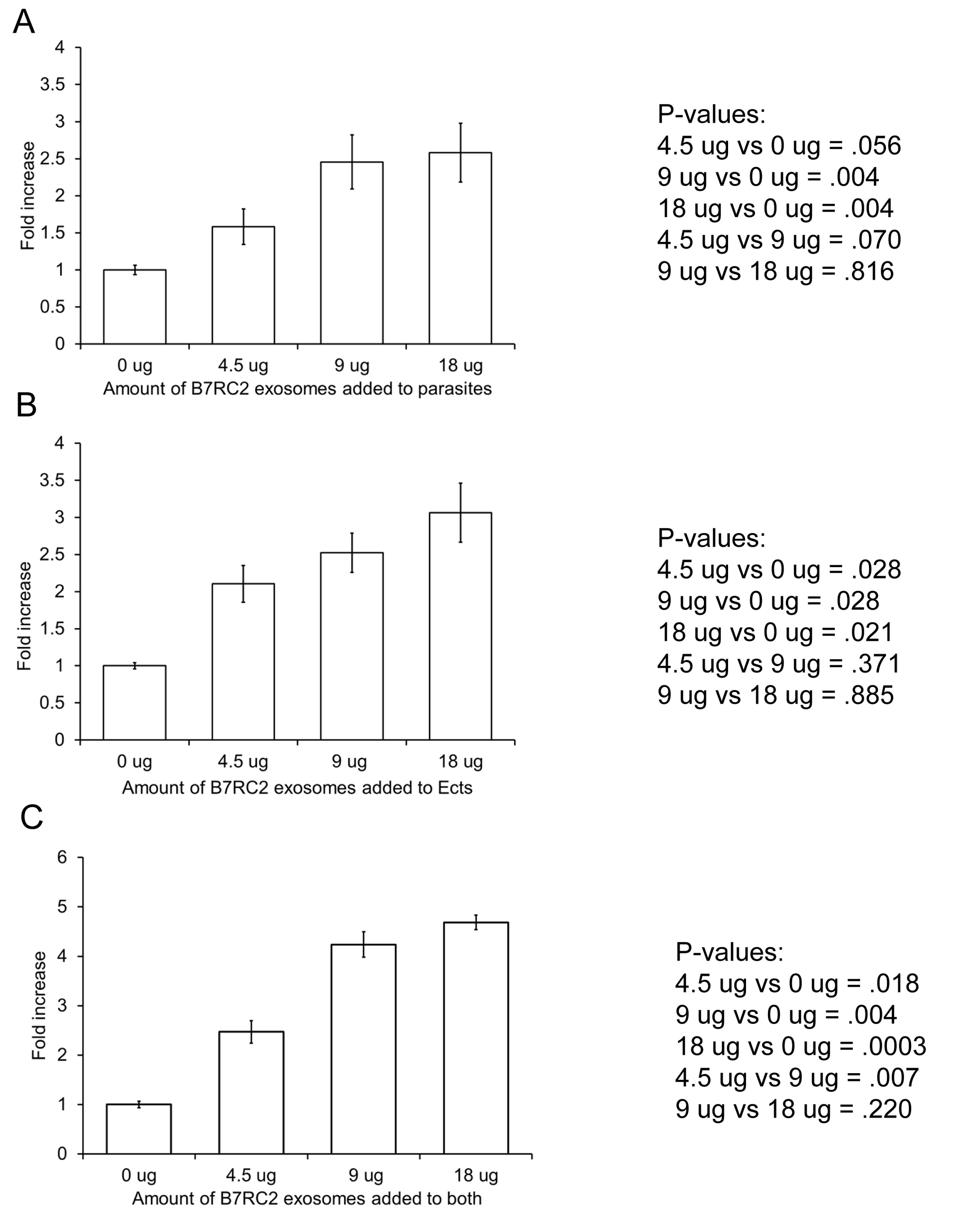

Supplement: Figure S2 — Exosomes increase parasite attachment to Ects in a dose dependent manner. Poorly adherent parasite strain G3 (A), Ects (B) or both (C), were preincubated with 0, 4.5, 9, or 18 ug of exosomes from the highly adherent B7RC2 strain, followed by washing to remove exosomes. Adherence of G3 parasites to the Ects was then measured. BSA preincubation of Ects (indicated as BSA) served as a negative control and was used to normalize experiments. The mean of three independent experiments each done in triplicate is shown ± SEM. (TIF) [file ppat.1003482.s002.tif]
